# Amyloid-β Acts as a Regulator of Neurotransmitter Release Disrupting the Interaction between Synaptophysin and VAMP2

**DOI:** 10.1371/journal.pone.0043201

**Published:** 2012-08-15

**Authors:** Claire L. Russell, Sophia Semerdjieva, Ruth M. Empson, Brian M. Austen, Philip W. Beesley, Pavlos Alifragis

**Affiliations:** 1 School of Biological Sciences, Royal Holloway University London, Surrey, United Kingdom; 2 Department of Physiology, University of Otago School of Medical Sciences, Dunedin, New Zealand; 3 Neurodegeneration Unit, Basic Medical Sciences, St George’s, University of London, Cranmer Terrace, London, United Kingdom; Federal University of Rio de Janeiro, Brazil

## Abstract

**Background:**

It is becoming increasingly evident that deficits in the cortex and hippocampus at early stages of dementia in Alzheimer’s disease (AD) are associated with synaptic damage caused by oligomers of the toxic amyloid-β peptide (Aβ42). However, the underlying molecular and cellular mechanisms behind these deficits are not fully understood. Here we provide evidence of a mechanism by which Aβ42 affects synaptic transmission regulating neurotransmitter release.

**Methodology/Findings:**

We first showed that application of 50 nM Aβ42 in cultured neurones is followed by its internalisation and translocation to synaptic contacts. Interestingly, our results demonstrate that with time, Aβ42 can be detected at the presynaptic terminals where it interacts with Synaptophysin. Furthermore, data from dissociated hippocampal neurons as well as biochemical data provide evidence that Aβ42 disrupts the complex formed between Synaptophysin and VAMP2 increasing the amount of primed vesicles and exocytosis. Finally, electrophysiology recordings in brain slices confirmed that Aβ42 affects baseline transmission.

**Conclusions/Significance:**

Our observations provide a necessary and timely insight into cellular mechanisms that underlie the initial pathological events that lead to synaptic dysfunction in Alzheimer’s disease. Our results demonstrate a new mechanism by which Aβ42 affects synaptic activity.

## Introduction

Alzheimer’s disease (AD) is a progressive neurodegenerative disorder. The brain of AD patients is characterised by neuronal loss, the presence of extracellular senile plaques comprised of β-amyloid peptide (Aβ) and intracellular neurofibrillary tangles (NFT) consisting of aggregates of hyperphosphorylated tau protein [1]. Aβ is derived from the proteolytic cleavage of the amyloid precursor protein (APP) [2] and the identification of Aβ as the major component of senile plaques led to the hypothesis that its extracellular deposition could be a key factor in the progression of AD [3]. Despite a clear association between Aβ build up and cognitive decline, [4], [5], [6], [7], [8] a correlation between plaque deposition and the severity of dementia, could not be established. On the contrary, the cognitive decline appears to be underlined by defects in synaptic plasticity and by loss or dysfunction of synapses [9], [10], [11] that precede Aβ deposition and NFT formation [12], [13], [14]. It is now believed that small soluble Aβ oligomers, are responsible for early synaptic changes [15].

Although it is well established that Aβ affects long term potentiation (LTP) and long term depression (LTD), the causal mechanisms are still elusive [16]. NMDAR dependent LTP in the hippocampus is blocked upon application of Aβ [7], [17], [18]. Intriguingly though, at low concentrations Aβ induces LTP possibly through α-7 nicotinic acetylcholine receptors [19]. Furthermore, Aβ induces LTD and excitotoxicity mediated by NMDARs receptors [20]. The importance of glutamate signalling via NMDARs as a causative event of dementia in AD is further demonstrated, by findings that memantine -a low affinity antagonist for NMDARs- results in behavioural improvement in AD model transgenic mice and is used as treatment of moderate AD [21], [22], [23]. This raises the possibility that the effects of Aβ could be due to an agonist action on NMDARs [24]. However, it is not fully established whether these effects are mediated directly through NMDARs. On the contrary evidence suggests that high concentration or prolonged exposure to Aβ42 is required to establish a direct effect on AMPA or NMDA receptors [10], [25], [26], [27]. Thus, it is unlikely that AMPA- and NMDA-receptors are directly affected at the earliest stages of AD pathology. Since there is no conclusive evidence of a direct interaction between Aβ and NMDARs, proposals such as a reduction in glutamate uptake or an increase of glutamate release have been put forward to explain these findings [19], [28], [29], [30], [31], [32], [33], but the cellular mechanisms underlying these defects are not clearly understood.

**Figure 1:**
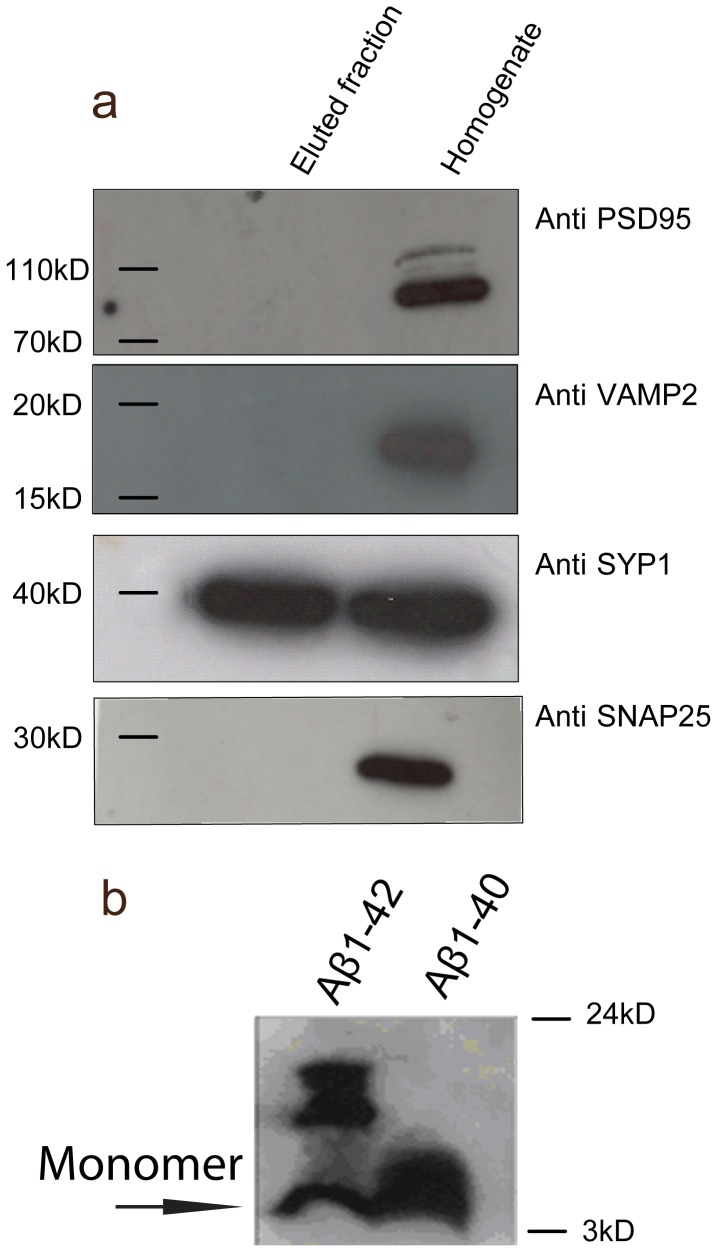
Identification of proteins interacting with Aβ by candidate approach. (a) Western blotting with primary antibodies against synaptic markers detected specific proteins in the starting material at their expected molecular weights. Only the primary antibody against Syp detected a specific protein in the fraction eluted from the Aβ affinity column. (b) Oligomerisation state of Aβ42 compared to Aβ40 revealed by western blotting. Monomeric Aβ peptides can be seen as a band at 4 kDa. Only Aβ42 displayed visible low molecular weight aggregates.

Here, we investigated cellular and molecular mechanisms by which Aβ induces synaptic toxicity. We show that administration of Aβ42 peptides to mature hippocampal neurons is followed by its internalisation. Subsequently, Aβ42 is detected at presynaptic terminals, where it can interact with Synaptophysin (Syp). We show that this interaction disrupts the Syp/VAMP2 complex and that this disruption could contribute to an expansion of the primed synaptic vesicle pool and of baseline neurotransmission.

## Materials and Methods

### Hippocampal Cell Culture

All animal experiments were performed according to Home Office regulations in appliance with the Animals Scientific Act 1986. Primary cultures of CA3-CA1 hippocampal neurons were prepared from E18 Sprague Dawley rat embryos. The experiments were performed in mature (21–28 days in vitro (DIV)) cultures. Neurons were seeded on poly-D-lysine (100 µg/ml in 0.1 M borate buffer) and laminin (5 µg/ml in PBS) coated coverslips at a density of 75,000 cells per coverslip and were maintained at 37°C, 5% CO_2_ in Neurobasal media, supplemented with B27, L-glutamine (0.5 mM) and 100 units/ml penicillin/streptomycin.

**Figure 2:**
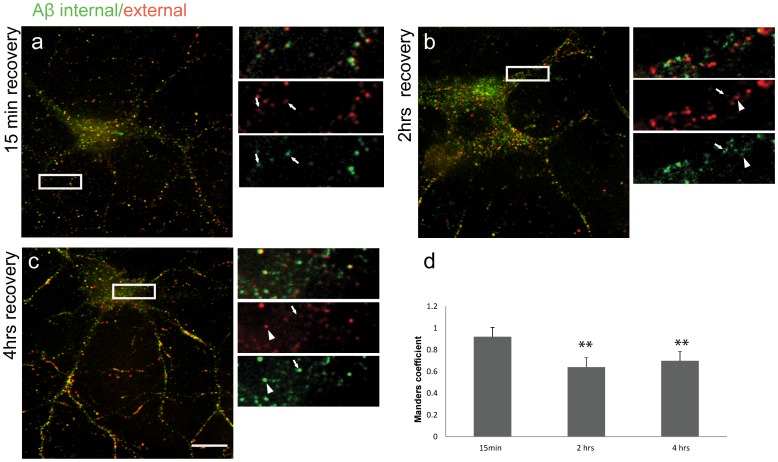
Representative images showing the dynamics of Aβ42 internalisation by sequential immuno-labelling. Mature hippocampal neurons incubated with Aβ42 for 20 min were left to recover for 15 min, 2 hrs and 4 hrs. (**a**) Labelling for external (red) and internalised (green) Aβ42 after 15 min recovery period. Arrows at high magnification images of boxed area (right) point at examples of overlapping staining. A reduction in co-labelling was observed after 2 hrs (**b**) or 4 hrs (**c**) recovery. Arrowheads at high magnification images of boxed area (right) show examples of external and arrows of internalised Aβ42. (**d**) Quantification of the extent of co-localisation shows a high degree of co-localisation at 15 min recovery (M = 0.92±0.07, n = 6) compared to 2 hrs (M = 0.64±0.07, student t-test ** = 0.0006, n = 10) and 4 hrs (M = 0.70±0.08, student t-test * = 0.0021, n = 8) of recovery. Scale bar = 10 µm.

### Immunocytochemistry

Hippocampal cultures were rinsed once with PBS and fixed with 4% paraformaldehyde (PFA) in PBS. Fixed neurons were washed, permeabilised with 0.1% Tween-20 and 5% horse serum in PBS for 45 min at room temperature, and were incubated with primary antibodies overnight at 4°C. After washing, cells were incubated for two hours at room temperature with Alexa Fluor 488 or Alexa Fluor 555 (Molecular Probes, UK). Primary antibodies used were: Anti-Aβ 6E10 (ID Labs Ontario, Canada and Abcam Cambridge, UK), Anti-NR2A/B (Millipore Watford, UK). All other primary antibodies were supplied by Abcam. For internalisation assays, fixed cells were incubated with 6E10 followed by Alexa Fluor 488, then postfixed in 4% PFA for two minutes. Cells were re-incubated with 6E10 in the presence of 0.1% Tween-20, followed by the secondary antibody Alexa Fluor 555. In all experiments, the cells were rinsed, mounted with ProLong reagent, and visualised on a spin disc confocal system (CARV from Digital Imaging Solutions) with an EM-CCD camera (Rolera/QI Cam 3500) mounted in an Olympus X71 microscope with a 100× objective, using Image Pro 6.0 software. High magnification inlets were produced using Adobe Photoshop.

**Figure 3:**
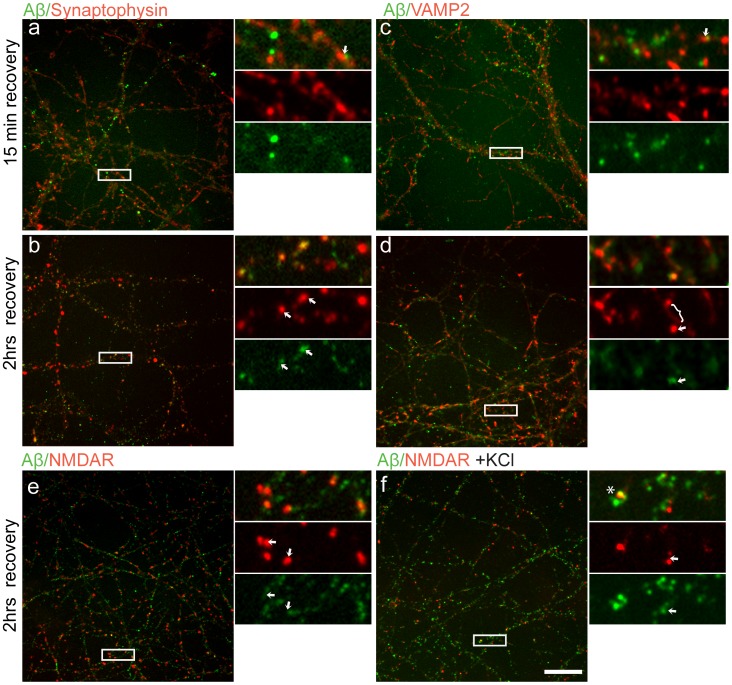
Representative images demonstrating presynaptic localisation of Αβ42 (green) in mature hippocampal neurons, incubated with Aβ42 for 20 min and left to recover for various time points. After 15 min recovery, Aβ42 and Syp (**a**) or Aβ42 and VAMP2 (**c**) labelling is distinct and often juxtaposed (as detailed in high magnifications of boxed areas (right), n = 15). After 2 hrs of recovery co-localisation of Aβ42 with Syp (**b**) and VAMP2 (**d**) can be observed (n = 39 for each condition). Arrows at high magnification images of boxed area (right) point at examples of Syp (b) or VAMP2 (d) positive synaptic contacts that are also positive for Αβ42. Bracket in high magnification images of boxed area (d) shows diffuse VAMP2 staining evident after the 2 hrs recovery period. After 2 hrs of recovery Aβ42 is often juxtaposed to NMDAR (**e**) (n = 18) even after depolarisation (**f**) (n = 10). Arrows in high magnification images of boxed area point at examples of Αβ42 juxtaposed to NMDAR. Scale bar = 10 µm.

### FM1-43FX Labelling

Following exposure to 50 nM of Aβ for 20 min and 2 hrs of recovery, 5 µM of FM1-43FX dye (prepared in HBSS warmed to 37°C) was applied to the cells for 5 min, followed by application of a hypertonic 500 mM sucrose solution in HBSS with 5 µM FM1-43FX. After 5 min the sucrose solution was replaced with the original FM1-43FX solution for 15 min. The cells were then washed twice for 15 min in HBSS to remove un-incorporated dye and were subsequently fixed in 4% paraformaldehyde. Cells were kept in the dark until utilised.

### Protein Extracts and Immunoprecipitation

Hippocampi were isolated from the brains of two juvenile Sprague Dawley rats of either sex (weighing between 90 g and 140 g) and homogenized in 1 mL lysis buffer (25 mM HEPES pH 7.5, 150 mM NaCl, 1% NP-40, 10 mM MgCl_2_, 1 mM EDTA, 2% Glycerol), containing a protease cocktail inhibitor (Roche, UK). Protein G Dynabeads® (25 µL) (Invitrogen, UK) were incubated with 5 µg of the precipitating monoclonal antibody for one hour at 4°C. Scrambled peptide and Aβ42 were diluted in 100 µl hippocampal protein extracts (∼7 mg/ml) at a final concentration of 100 µM and the mix was incubated with the antibody bound Dynabeads® at 4°C for 4 hrs. Samples were boiled for 5 min to elute bound proteins, which were then analysed by SDS-PAGE.

**Figure 4:**
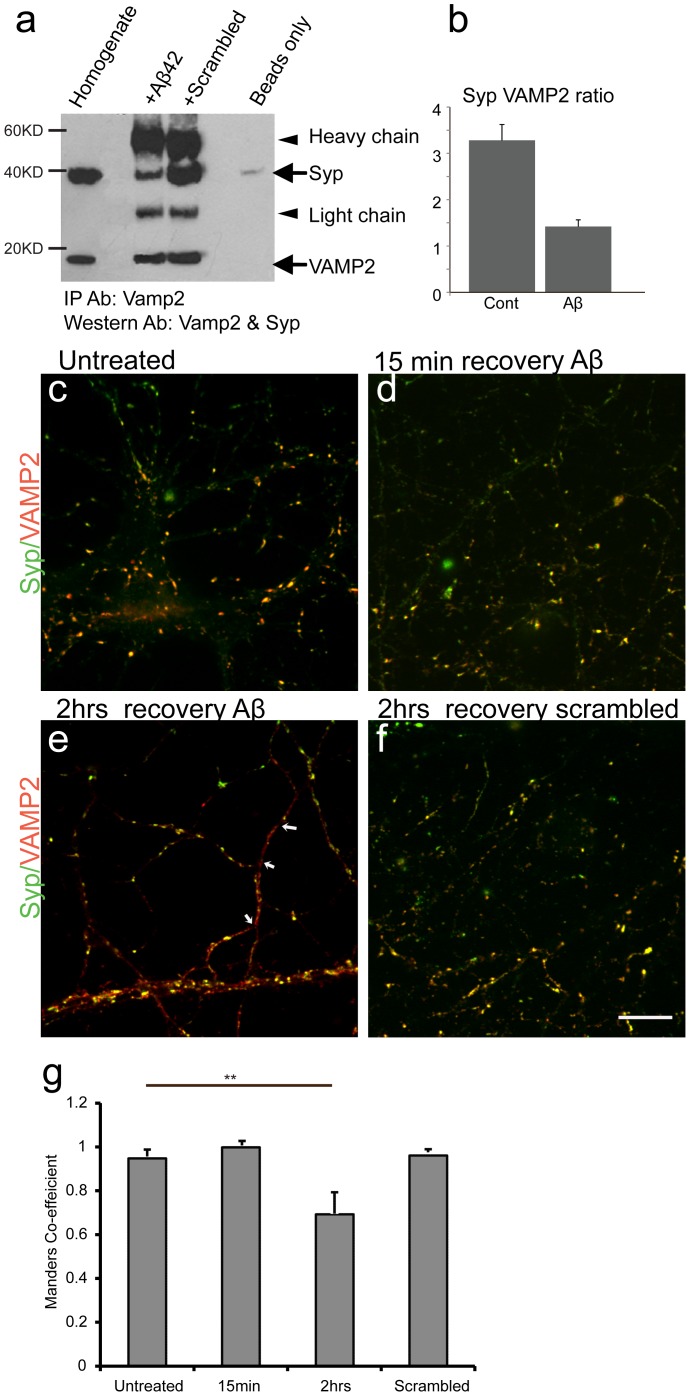
Disruption of the Syp/VAMP2 complex. (**a**) Immunoprecipitation of VAMP2 from hippocampal extracts assayed by western blotting for VAMP2 and Syp. A band of 19 kDa corresponding to VAMP2 and a band of 38 kDa, corresponding to Syp were detected in all conditions. A reduction in immunoprecipitated Syp was induced by Aβ42 (lane 3) compared to control (scrambled Aβ, lane 4). (**b**) The intensity ratio between Syp and VAMP2 is reduced in the presence of Aβ42 (n = 3, student t-test p = 0.0026). (**c**) co-labelling for Syp and VAMP2 in untreated hippocampal neurons, in neurons treated either with Aβ42 and allowed to recover for 15 min (**d**) or with scrambled peptide (**f**) display a significant degree of co-localisation. (**e**) Aβ42 treated neurons allowed to recover for 2 hrs display stretches of VAMP2 immunoreactivity (indicated by arrows). (**g**) Quantification of the extent of co-localisation between Syp and VAMP2. Aβ42 treated neurons left to recover for 2 hrs showed decreased level of co-localisation (M = 0.85±0.09, n = 24) compared to control (M = 0.961±0.03, n = 24, student t-test ** = 0.0003), whereas neurons left to recover for 15 min (M = 0.99±0.02, n = 24) or incubated with the scrambled peptide (M = 0.99±0.01, n = 18) did not display differences compared to control. Scale bar = 10 µm.

### Immunoblot

Proteins were separated on NuPage 4–12% Bis-Tris gels at 200 V and transferred to nitrocellulose membrane at 100 V for 1 hr at 4°C in transfer buffer (200 mM Glycine, 25 mM Tris Base 20% Methanol). Blots were blocked with 5% nonfat dry milk in Tris-buffered saline (50 mM Tris Base, 150 mM NaCl) containing 0.1% Tween 20, pH 7.5, for 1 hr and incubated overnight at 4°C with primary antibody (1∶250) and 2 hrs with HRP-conjugated secondary antibody (1∶1000). Membranes were developed with SuperSignal chemiluminescence kit according to instructions.

**Figure 5:**
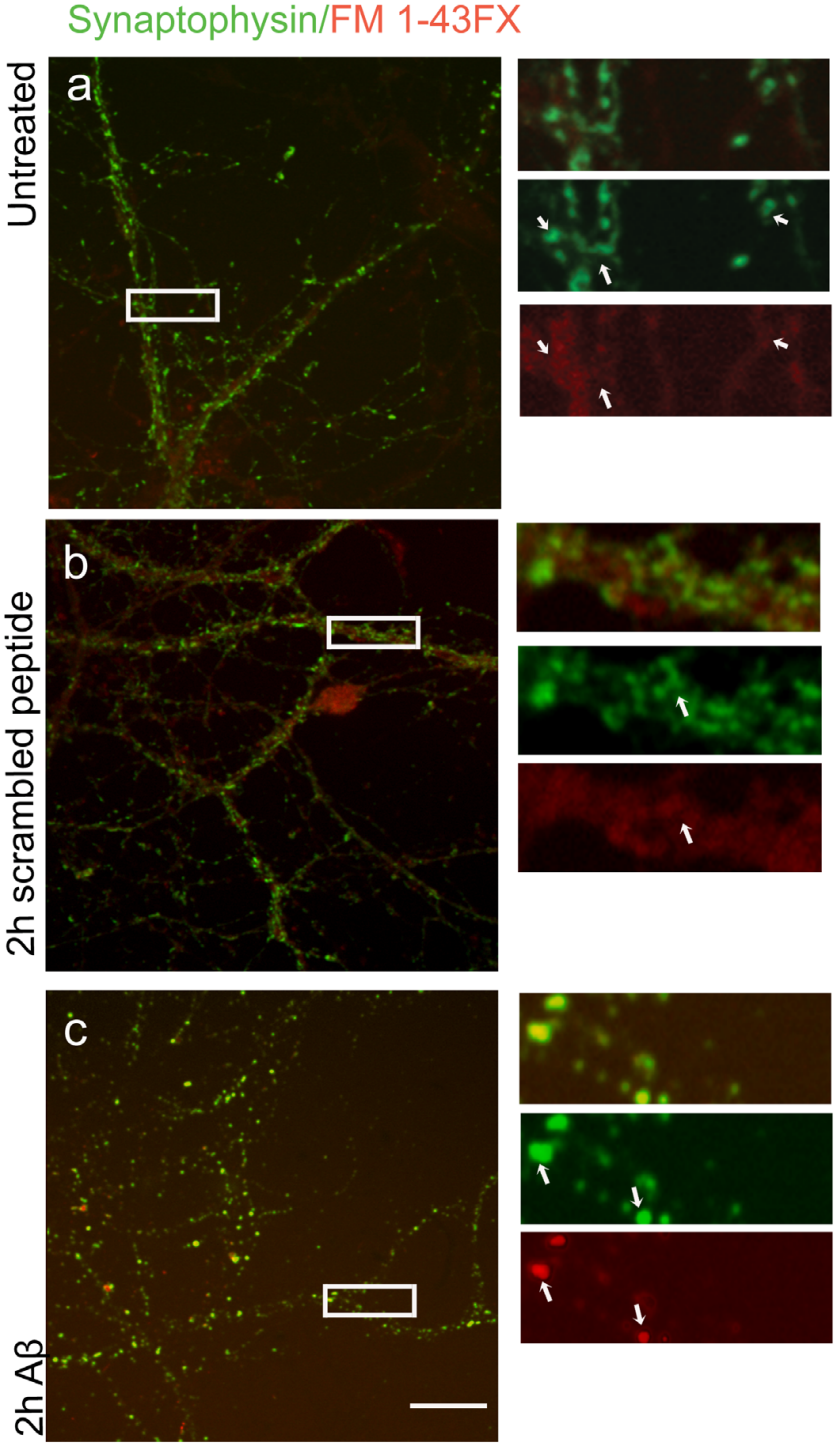
Fusion of primed vesicles at synaptic contacts marked by labelling for Syp (green) is visualised by the extent of internalisation of FM1-43FX dye (red). High magnification images of boxed areas are shown on the right. Internalised dye at synapses is near background levels in untreated (n = 20) (**a**) as well as in neurons treated with the scrambled peptide (n = 20) (**b**). Arrows in a and b point at synaptic contacts labelled with Syp. (**c**) Exposure to Aβ42 followed by 2 hrs of recovery results in a significant increase in internalised dye at synaptic contacts (n = 20). Arrows in c point at synaptic contacts labelled with Syp. Scale bar = 10 µm.

**Figure 6:**
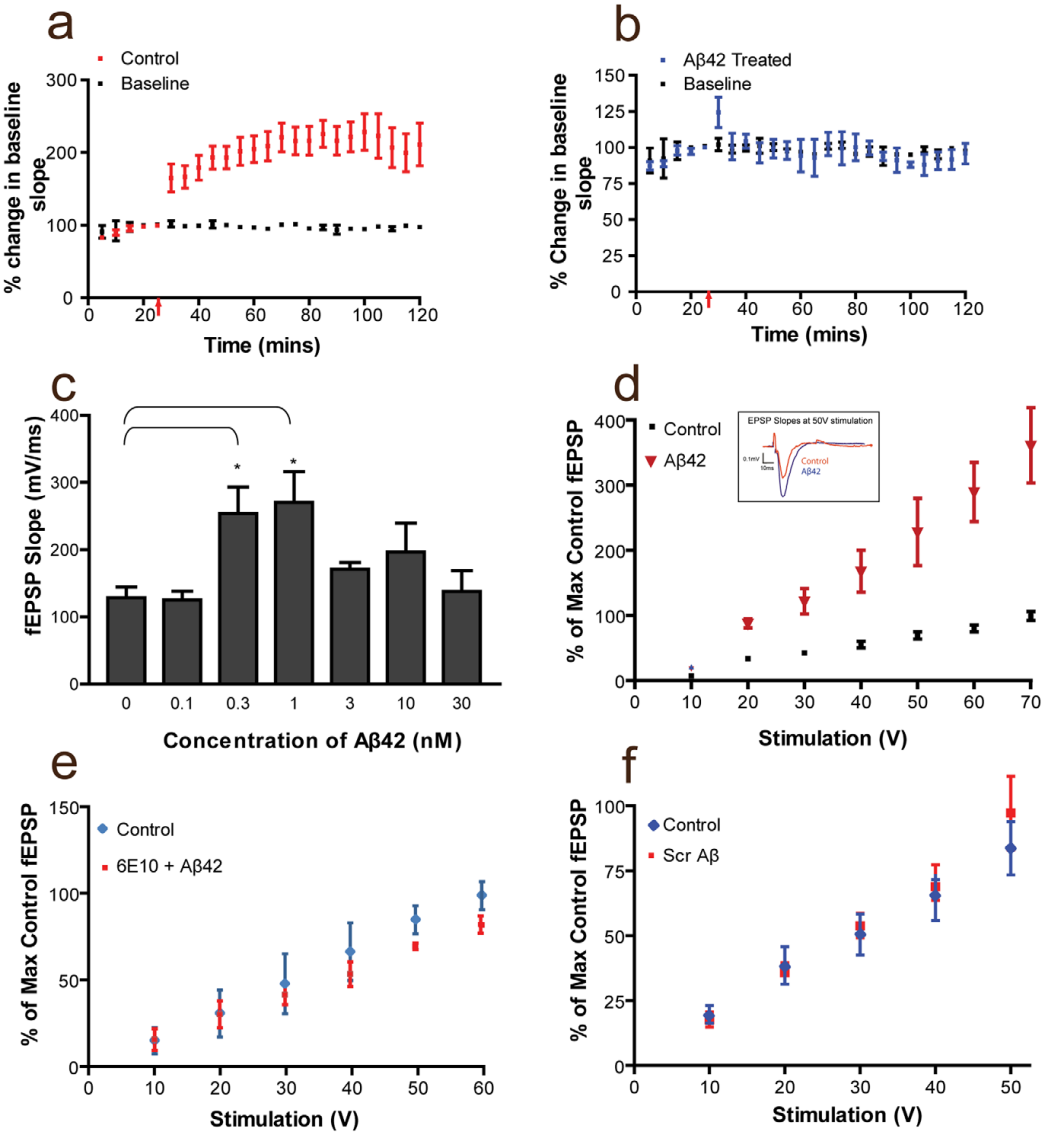
Neurotrasmitter release is affected by Aβ. (**a**) Induction of CA1- LTP after HFS in control hippocampal slices (Two-way ANOVA p = 0.0006 n = 5). (**b**) Induction of CA1-LTP after HFS is blocked in hippocampal slices after a 45 min bath application of Aβ42. (**c**) fEPSPs recorded in the CA1 in response to a 50 V stimulus of the Schaffer collateral commissural pathway showed a concentration dependent effect of Aβ in fEPSPs. Only mid-range concentrations of 0.3 nM and 1 nM were able to increase the fEPSP slope (p = 0.028 and p = 0.048 respectively with student t-test, n = 5 for each concentration tested). (**d**) Treatment with Aβ42 (45 min) increases the stimulation response compared to control, untreated slices over a range of stimuli. Results are presented as the percentage of the maximum fEPSP from control recordings (n≥5 each). Inlet in shows a trace of EPSP slopes between a control slice and a slice treated with Aβ42 stimulated at 50 V. (**e**) Chelation of Aβ42 using 6E10, a specific antibody for the peptide, abrogates the enhancement at all input values (n = 5). (**f**) Scrambled Aβ did not induce increased responses compared to controls.

### Hippocampal Slice Preparation

Field potential recordings were made from ventral sections of postnatal day 32–42 Sprague Dawley rats of either sex. Rats were deeply anesthetised by halothane prior to decapitation, and the brain rapidly removed and submerged in oxygenated (95% O_2_, 5%CO_2_) artificial cerebrospinal fluid (aCSF) containing 135 mM NaCl, 3 mM KCl, 1.25 mM NaH_2_PO_4_, 1 mM MgCl_2_, 10 mM Glucose and 26 mM NaHCO_3_. The brain was hemisected, and ventral sections (400 µm thick) prepared in aCSF on a Vibroslice (Campden Instruments Ltd). Slices were transferred and submerged in a holding chamber of continually oxygenated aCSF and left to recover for at least one hour. Aβ42, Aβ40 and scrambled Aβ peptide were added to the chamber to treat slices for 45 minutes prior to field EPSP recordings. Lyophilised fraction from 7PA2 cell media containing nanomolar amounts of Aβ oligomers was re-suspended in 100 ml aCSF in the tissue chamber to treat hippocampal slices for 45 min.

**Figure 7:**
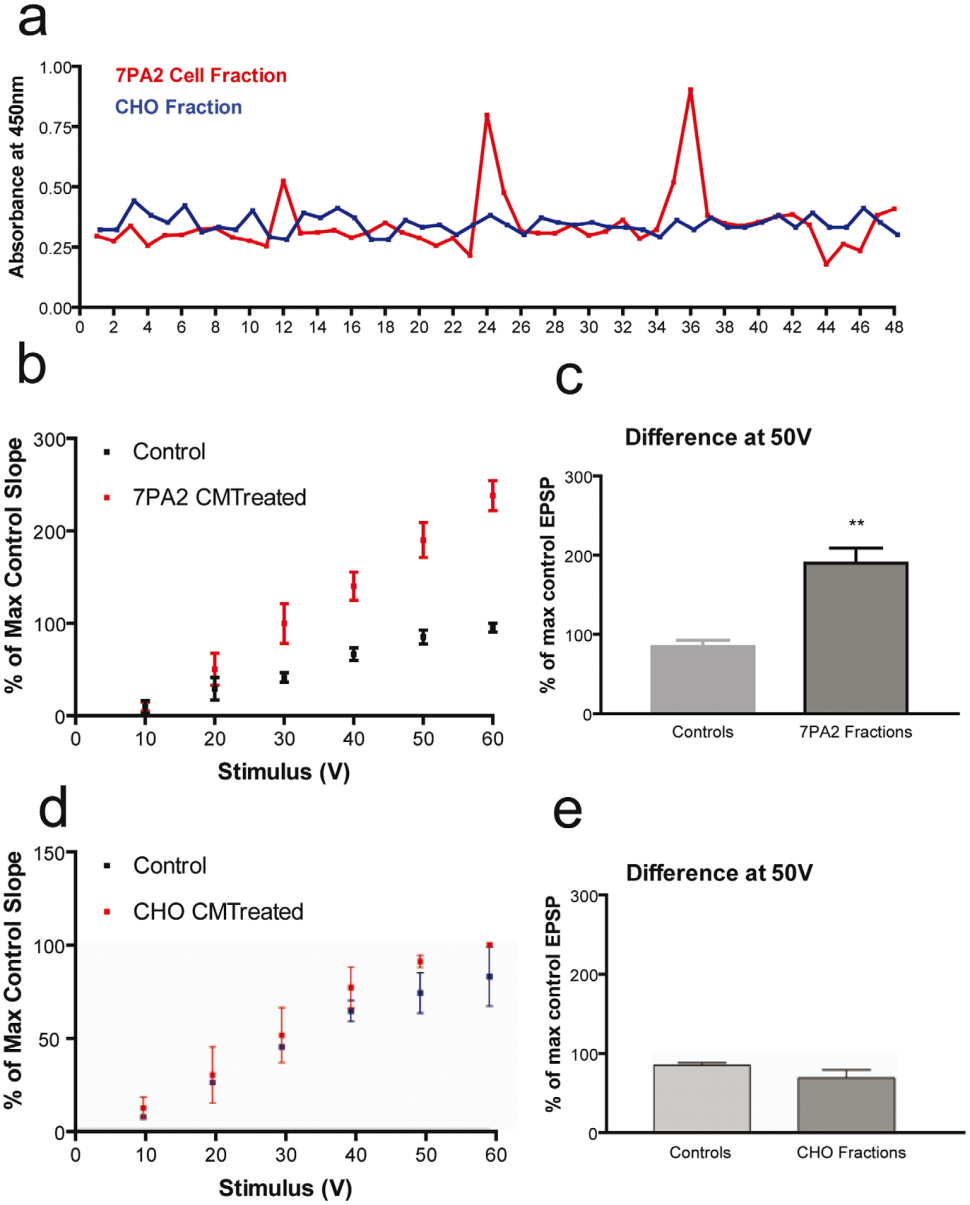
ELISA assay from fractionated conditioned media (CM) from 7PA2 cells and control CHO cells detected Aβ oligomers in the conditioned medium of 7PA2 cells but not of CHO control cells (a). Hippocampal slices were incubated with CM from fractions 12, 24 and 36 of control as well as 7PA2 cells. fEPSPs were recorded in response to Schaffer collateral commissural pathway stimulation at a range of stimulus from 10 V through to 60 V (b and d). Incubation with cell-derived Aβ, increased the response to stimulation at the CA1 synapse (**b**). compared to slices treated with conditioned media from the control CHO cells (**d**). The results are expressed as a percentage of the maximum fEPSP slope from control slices. Graphs showing representative results at 50 V in slices exposed to 7 PA conditioned medium (**c**) and to control CHO conditioned medium (**e**) show that cell derived Aβ increased fEPSP (student’s t-test p = 0.003 n = 5), whereas the control medium had no affect at 50 V (n = 5) (e).

### Electrophysiology

Recordings of field excitatory post synaptic potentials (fEPSPs) were made from the stratum radiatum in the CA1 region of the hippocampus using low resistance glass electrodes, in response to stimulation of the Schaffer collateral commissural pathway using a monopolar borosilicate glass electrode driven by a constant voltage stimulator (Digitimer) DC recordings were amplified using an AxoClamp2A (Molecular Devices) and a Neurolog NL104 (Digitimer Ltd) and signals filtered at 3 kHz prior to digitisation with a CED1401. Experiments were controlled using Signal 2 (CED Ltd) running on a connected PC. Electrodes were positioned just below the tissue surface (liquid interface setup) and slices were left to stabilise for 20 min. Input/output curves (or stimulation responses) were recorded, and the 10–90% slope measured, from a stimulus ranging from 10 V to 70 V with 3 successive events at each input level, 30 sec apart. To detect concentration dependent changes in neurotransmitter release, fEPSPs were initially evoked at 50 V with low frequency at 0.033 Hz in the presence of different concentrations of Aβ42. For LTP recordings, a stable baseline was maintained for 25 min at 50% of the maximum fEPSP response, and LTP was induced by high frequency stimulation (HFS) consisting of three trains of 100 Hz, 10 sec apart. Potentiated synaptic response was subsequently recorded with low frequency stimulation every 30 sec for at least 90 min.

### Synthetic Aβ Peptides

Aβ42 (American Peptide USA) was prepared as described previously [34] and stored in 0.1 M Tris pH7.4 at a stock concentration of 100 µM at −20°C. Neurons were incubated with 50 nM of peptide for 20 min. Following treatment, the media was replaced with fresh supplemented Neurobasal and the cells were left to recover for the specified times.

### Cell-Derived Aβ

7PA2 cells (a kind gift from D.M. Walsh, University College Dublin) [35], are a CHO cell line stably expressing human APP_751_ carrying the V717F AD causing mutation. Cell cultures of 90% confluency were washed with serum free DMEM followed by incubation for 18 hrs in serum free DMEM. The conditioned medium was collected, cleared of cells by centrifugation (134×g_av_ for 10 mins), and protease inhibitors were added to the supernatant. Aβ oligomers were separated by size exclusion chromatography as described [36]. Briefly, 1 ml of concentrated CM from each cell line was injected onto a Superdex 75 10/30 HR column attached to a AKTA HPLC system (Amersham) and eluted at a 0.4 mL/min. flow rate with 50 mM ammonium acetate (pH 8.5). The eluent was monitored at 280 nm and 230 nm and collected in fractions (0.5 ml). 50 µl of each fraction was used in a double ELISA assay to determine Aβ oligomer content as previously described [37]. The remaining fractions containing Aβ peptides were pooled together and used to treat hippocampal slices.

## Results

### Aβ Interacts with Synaptophysin

To examine the underlying mechanisms behind Aβ42 induced excitotoxicity, we designed a proteomic screen, based on an affinity column of Aβ42, to identify synaptic proteins from hippocampal extracts that bind to the peptide. Bound proteins were first characterised by a candidate approach using antibodies against specific synaptic proteins to probe Western blots. Among various proteins examined, Syp, a protein present at synaptic vesicles (SV) was detected at high levels (fig. 1a). Antibodies against additional pre-synaptic markers, such as synaptosomal-associated protein 25 (SNAP25) and vesicle associated protein 2 (VAMP2 or synaptopbrevin 2) failed to detect any protein in the eluted fractions. In addition, antibodies against postsynaptic proteins, such as the post synaptic density protein PSD95 were also used and again no bands were observed (fig. 1a). These results suggest that Syp is a strong candidate to be a specific interacting partner of Aβ42.

### Internalisation of Aβ42 in Neurons

We then explored the significance of this interaction in dissociated neurons. For this interaction to occur *in vivo*, Aβ42 should be present inside the neurons, at the presynaptic terminal. Aβ42 accumulates intracellularly via two possible avenues; either it builds up because part of the intracellular pool is not secreted, or extracellular Aβ42 is internalised by neurons [38]. Thus, we developed an internalisation assay to investigate the dynamics and the subcellular distribution of externally administered synthetic Aβ42. Synthetic Aβ42 is commercially available and readily aggregates in aqueous solution [34]. Since soluble Aβ42 oligomers are believed to be the pathogenic peptides responsible for synaptic changes [36], we first ensured that oligomers were indeed present in our preparations, examining the aggregation state of Aβ42 by Western blotting. Oligomeric as well as monomeric Aβ42 species were consistently detected (fig. 1b). We then established the optimum conditions that would allow detection of the peptide via immuno-labelling and also ensure optimum survival of the neurons, applying different concentrations of Aβ42 to mature (21 DIV) dissociated hippocampal neurons.

Our results showed that incubation of dissociated hippocampal neurons with 50 nM of Aβ42 for 20 min, followed by recovery periods of 15 min, 2 hrs and 4 hrs allowed both, detection of Aβ42 and optimum survival of neurons. To look into the dynamics of the internalisation of Aβ42, sequential immuno-labelling of fixed neurons (treated with 50 nM of Aβ42) was employed to distinguish between surface and internalised Aβ42. Surface labelling was carried out using the 6E10 primary antibody (for Aβ detection) and the Alexa-Fluor 488 secondary antibody in the absence of detergent. The neurons were then post fixed and intracellular Aβ42 was detected by repeating the procedure using the same primary antibody and the Alexa-Fluor 555 secondary antibody in the presence of 0.1% Tween-20 to permeabilise the cells. After 15 min of recovery, a high degree of co-labelling was observed, suggesting the peptide remained extracellular (fig. 2a). However, with increased recovery time, although co-labelling was still evident, additional signal was increasingly observed after permeabilisation (fig. 2b, c) showing that after 2 hrs of recovery Aβ42 had been internalised. To quantify the extent of the internalisation, the co-localisation was quantified by calculating the split Mander’s co-localisation coefficients using the JACoP plugin for Image-J [39]. Mander’s co-efficient measures the coincidence of two signals, even when the intensities in both channels are significantly different. Our results (fig. 2d) show that the Mander’s coefficient was highest (0.92±0.7 *n = 6*) after the 15 min recovery time point, suggesting a high degree of co-localisation. Within a 2 hrs recovery period, the Mander’s coefficient dropped significantly (0.64±0.07 *n = 10, student t-test p = 0.0006*). Similarly, after 4 hrs of recovery, the Mander’s coefficient was lower than the 15 min recovery period (0.70±0.08 *n = 8, student t-test p = 0.0021*) confirming our observation that Aβ42 can be detected intracellularly over a 4 h period [40].

### Presynaptic Localisation of Aβ42

Since we identified Syp as a possible interacting partner of Aβ42, we examined if such an interaction could mediate the internalisation/endocytosis of Aβ42 at synaptic contacts. Dissociated neurons were incubated with Aβ42 (50 nM) for 20 min and allowed to recover in fresh media for 15 min or 2 hrs prior to fixation. If Aβ42 was internalised via its interaction with Syp, we would expect Aβ42 to be localised at presynaptic terminals soon after incubation with the peptide. Double immuno-labelling for Aβ42 and Syp after 15 min of recovery, did not reveal any significant overlap between Aβ42 and presynaptic contacts marked by Syp labelling (fig. 3a, n = 15). On the contrary, Aβ labelling was often detected juxtaposed to Syp (arrow in high magnification of fig. 3a) suggesting a postsynaptic localisation of Aβ [11], [41]. After 2 hrs of recovery however, co-localisation between Aβ42 and Syp labelling in a fraction of synapses was evident (fig. 3b, n = 39). These results showed that although internalisation of Aβ42 via an interaction with Syp is unlikely, the peptide can be detected pre-synaptically after an extended period of time. To further confirm our findings, we repeated the experiment using VAMP2 as an alternative presynaptic marker. Similar to our previous observations, after 15 min of recovery no overlapping pattern between Aβ42 and VAMP2 labelling was evident (fig. 3c, n = 15), whereas after 2 hrs of recovery, co-localisation between VAMP2 and Aβ42 could be detected (fig. 3d, n = 39), further confirming its presence at pre-synaptic terminals. However, compared to Syp, the pattern of VAMP2 staining after 2 hrs of recovery was unusual, with visible stretches along the membrane (see brackets in high magnification of fig. 3d) instead of the characteristic punctate pattern (fig. 3c). Finally, double immuno-labelling for Aβ42 and NMDARs in dissociated neurons incubated with 50 nM of Aβ42 for 20 min and allowed to recover in fresh media for 2 hrs prior to fixation showed that Aβ42 could be detected alongside as well as juxtaposed to NMDARs confirming its presynaptic localisation (arrows in fig. 3e). However, these data were unexpected since previous reports have demonstrated that incubation of dissociated neurons with Aβ peptides results in its postsynaptic localisation [11], [41], [42]. Since neurotransmitter release has been associated with a rapid increase in the extracellular levels of Aβ42 [43], we considered the possibility that the observed presynaptic localisation of Aβ42 could be transient, followed by its release into the synaptic cleft in an activity dependent manner. Subsequently the released Aβ42 would interact with postsynaptic targets. To test this hypothesis, dissociated hippocampal neurons exposed to 50 nM of Aβ were depolarised after 2 hrs of recovery. The neurons were treated with 50 mM KCl for 10 min to induce neurotransmitter release. Double immuno-labelling for Aβ42 and NMDARs showed that a substantial amount of Aβ42 was still juxtaposed to, NMDARs (fig. 3f). Quantification of the extent of co-localization between Aβ and NMDARs by calculating the Mander’s co-efficient, did not reveal any statistically significant differences between neurons exposed to Aβ42 (0.296±0.05) and neurons exposed to Aβ42 and depolarised after recovery (0.348±0.03, student t-test p = 0.098, n = 10). These results suggest that the presynaptic localisation of Aβ42 was not transient since a substantial amount of the peptide remained presynaptic even after induction of neuronal activity.

### Aβ42 Disrupts the Syp/VAMP2 Complex

The biochemical interaction of Aβ42 with Syp and its presence at the presynaptic terminal suggests that the peptide may have a pre-synaptic effect. To explore this further, we looked closer at the unusual distribution of VAMP2 in neurons exposed to Aβ42 (high magnification of fig. 3 d). It has been shown that Syp interacts with VAMP2 regulating its synaptic distribution [44]. Thus we hypothesised that the diffuse distribution of VAMP2 could be a consequence of Aβ42 interfering with the formation of the VAMP2/Syp complex. To test this, we immunoprecipitated VAMP2 complexes from hippocampal extracts either in the presence of an excess of Aβ42, or in the presence of a similar concentration of control, scrambled Aβ (fig. 4a). A substantial reduction of co-immunoprecipitated Syp was observed in the presence of Aβ42 (fig. 4b, n = 3), compared to the amount of co-immunoprecipitated Syp in the presence of the scrambled peptide suggesting that Aβ42 disrupts the interaction between VAMP2 and Syp in vitro.

Since it has been shown that disruption of the interaction between VAMP2 and Syp induces the axonal distribution of VAMP2 [44], we compared the extent of co-localisation between these two proteins in neurons exposed to Aβ42 and control neurons to investigate if Aβ42 disrupts their interaction in living neurons as well. Double immuno-labelling of VAMP2 and Syp in control neurons (fig. 4c) and in neurons recovering for 15 min after exposure to 50 nM of Aβ42 (fig. 4d) showed the expected extensive co-localisation between the two proteins suggesting that Aβ42 had little or no effect on the VAMP2-Syp complex during the first 15 min of recovery. On the contrary, VAMP2 labelling in neurons exposed to Aβ42 after 2 hrs of recovery (fig. 4e) showed stretches of VAMP2 immunoreactivity devoid of Syp labelling (i.e. areas between arrows in figure 4e). This diffuse VAMP2 labelling is indicative of a disruption in the formation of the VAMP2/Syp complex *in vivo* [44] demonstrating that Aβ42 interferes with the stability of the interaction between VAMP2 and Syp. The co-localisation of VAMP2 and Syp was not affected in neurons exposed to similar concentration of the scrambled peptide (fig. 4f) suggesting the diffuse VAMP2 labelling is specifically induced by Aβ42 and not by the addition of any non specific peptide in the medium. Quantification of the extent of co-localisation between Vamp and Syp, by calculating the Mander’s co-efficient, confirmed that their co-localisation was specifically reduced in neurons exposed to Aβ42 and allowed to recover for 2 hrs (fig. 4g).

### Physiological Consequences in Synaptic Transmission

Further to its role in sorting VAMP2, Syp also interacts with VAMP2 at the presynaptic terminal and it has been proposed that it regulates the participation of VAMP2 in the SNARE complex during the formation of the fusion pore complex (FPC) [45], [46], [47]. Therefore, disruption of the association between VAMP2 and Syp at synaptic contacts by Aβ42 could induce the formation of the FPC resulting in an increase of primed SVs. To investigate this hypothesis, untreated mature hippocampal neurons or neurons treated for 15 min either with 50 nM of Aβ42 or with the inverted control peptide, were incubated after 2 hrs of recovery with FM1-43 FX, a fluorescent lipophilic dye that labels membranes. Using a hypertonic sucrose solution (500 mM sucrose in HBSS) primed vesicles were induced to fuse to the presynaptic membrane and neurons were left to recover in the presence of the dye to allow for its uptake at sites of SV recycling [48]. Incorporation of the dye at synaptic contacts was confirmed with immuno-labelling for Syp. Images of mature (21 DIV) untreated hippocampal neurons (fig. 5a) and neurons treated either with scrambled peptide as a negative control (fig. 5b) or with Aβ42 (fig. 5c) were taken consecutively leaving the microscope/camera settings unaltered. In untreated neurons or in neurons exposed to the control inverted peptide, uptake of the dye at synaptic contacts was near background levels, although internalisation of the dye was evident in the cell soma (n = 20, fig. 5 a, b). On the contrary, in neurons exposed to Aβ42, the dye uptake was more pronounced at a fraction of synaptic contacts (fig. 5c, n = 20), indicative of an increase in the number of vesicles primed to the presynaptic membrane. These results strongly suggest that Aβ42 increases the amount of primed vesicles at the presynaptic terminal. The question now was if this increase would contribute to synaptic transmission. Although our results show that Aβ42 could be detected at the presynapse via immuno-labelling after 2 hrs of recovery, it has been demonstrated that incubation of slices with Aβ42 for a shorter period of time, is necessary and sufficient to induce detectable synaptic defects [19], [49]. To account for possible differences in the physiology of neurons between hippocampal slices and dissociated neurons, we adopted a similar approach to investigate the effect of Aβ42 on synaptic transmission. Consistent with previous studies, hippocampal slices incubated with Aβ42 for 45 min prior to recording showed that Aβ42 inhibited the induction of NMDAR-dependent LTP in the hippocampal CA1 region (fig. 6a, b). Next, we investigated if under the same conditions Aβ42 affected basal transmission. We compared fEPSPs recorded from the CA1 region of hippocampal slices in response to electrical stimulation of the Schaffer collateral pathway, in the presence or absence of Aβ42. First, we noticed a concentration dependent enhancement in basic transmission at concentrations ranging from 300 pM to 1 nM Aβ42 (fig. 6c). We subsequently investigated this effect in detail by increasing electrical stimulation in slices exposed to 1 nM Aβ42. Our results showed a progressive enhancement of fEPSPs recorded in treated slices compared to untreated control slices (fig. 6d, p = 0.044 at 50 V with student’s t-test, n = 5), indicative of an increased efficacy of the Schaffer collateral synapse. We then confirmed the specificity of this enhanced efficacy either by chelation of Aβ42 using an excess of an Aβ42 specific antibody (200 ng/µl of 6E10 mAb), or by using equal concentrations of the scrambled peptide. Co-incubation with 6E10 and Aβ42 produced similar recordings to untreated slices (fig. 6e, n = 5). Furthermore, incubation of slices with the scrambled peptide had no effect (fig. 6f, n = 5) further confirming that the enhancement of fEPSPs was specific to Aβ42.

To unambiguously confirm that the enhancement of synaptic transmission was induced by Aβ, we repeated our experiments with cell-derived Aβ peptides, from 7PA2 cells, These Chinese-hamster ovary cells that express the V717F mutant human APP secrete low (nanomolar) levels of oligomeric Aβ peptides [35], [50]. Incubation of slices with cell derived oligomers provided similar results to those obtained with the synthetic peptide, suggesting that the enhancement of fEPSPs is not an artefact of the synthetic peptide (Fig. 7) but rather a physiological function of Aβ.

## Discussion

### Synaptic Localisation of Aβ42

Whilst extensive information in the literature shows that synaptic failure precedes cognitive decline in AD [4] the cellular and molecular events underlying this synaptic dysfunction are still not fully resolved. The data presented here provide an insight into one mechanism by which Aβ42 affects synaptic activity. First, we show that the presynaptic protein Syp interacts with Aβ42 *in vitro*. We then establish the significance of this interaction by showing that Syp and externally applied Aβ42 can co-localise in living neurons. Studies with transgenic animal models of AD, have supported the presence of intraneuronal Aβ (iAβ) prior to the appearance of extracellular deposits, suggesting that iAβ is a significant contributor to the onset of learning and memory deficits [38], [51]. Despite an apparent relationship between the intracellular pool of Aβ and the extracellular deposits [13], [52] the precise mechanism by which the peptide enters the neuron is still under investigation. Physical interaction between Aβ42 and the α7 acetylcholine receptor [53] or apoE [54] suggests that these proteins are candidates for a receptor mediated transfer of Aβ into the cell. On the other hand, there is evidence that Aβ peptides cross the neuronal membrane passively, via non-endocytic and energy independent pathways, most likely due to its ability to biophysically interact with lipids at the neuronal membrane [55]. Thus, we considered the possibility that an interaction between Syp and Aβ42 at the presynaptic terminal could be an alternative mechanism for its internalisation. We examined the dynamics of the co-localisation of these proteins and found no evidence to support the hypothesis that interaction of Aβ42 with Syp provides an alternative mechanism for the internalisation of the peptide, since there was no indication of presynaptic localisation of Aβ42 in neurons that were left to recover for only 15 min. Interestingly though, the absence of co-localisation at early time points compared to the co-localisation we observed 2 hrs after the removal of Aβ from our cultures, shows that Aβ42 can accumulate at the presynaptic terminal with time. It is therefore likely that, at least in part, the presynaptic localisation of Aβ42 could be due to its translocation at the presynaptic terminal by an unknown transport mechanism. Co-labelling for Aβ42 and NMDARs further confirmed that Aβ42 could be detected at presynaptic as well as at postsynaptic terminals.

In contrast to the data presented here, several reports demonstrate exclusive co-localisation between Aβ42 and postsynaptic markers [11], [41]. In one such study, Lacor and colleagues found 92% of exogenously applied Aβ co-localised with the post-synaptic protein PSD-95 and the majority of the peptide was found juxtaposed to Syp [41]. We believe that the difference in our results reflects differences in our experimental procedures. For instance, a plausible explanation is that we allowed cells to recover for two to four hours after exposure to Aβ42. This recovery period (in the absence of the peptide) proved to be essential for the detection of the peptide at presynaptic terminals. In addition, Lacor *et.al.* showed that only high molecular weight species of Aβ peptides could be detected post-synaptically whereas our Aβ preparation was enriched in low oligomeric forms. We suggest that taken together, these results complement each other, demonstrating two different roles of Aβ at the synapse, one presyanptic and the other postsynaptic.

### Mechanism of Synaptic Toxicity

Confusing data regarding the effect of Aβ42 in synaptic dysfunction such as increase or suppression of spontaneous activity are often found in the literature [56], [57]. The underlying problem in resolving these inconsistencies has been to understand the mechanisms by which differences in the amount/preparation of the peptides can affect the outcome of an experiment. Furthermore, the ambiguousness of the causal mechanisms behind these effects has further challenged consistent interpretation of these data. We believe that our findings regarding the presynaptic localisation, the interaction between Aβ42 and Syp and its effect in neurotransmitter release may help resolve some of these inconsistencies. Syp was the first SV protein to be characterised [58], [59]. Since then, various studies have implicated Syp in exocytosis, in regulating the formation of the fusion pore complex (FPC), in synapse formation and in endocytosis of SVs [45], [46], [47], [60], [61], [62], [63]. However, studies using Syp KO mice failed to show an apparent SV recycling phenotype [64], [65]. The lack of an obvious phenotype was attributed to the redundant function of its isoforms Synaptogyrin (syg) and Synaptoporin. Indeed, mice lacking both Syp and Syg expression showed reduced LTP [66] although the mechanism responsible for this reduction is still not clear.

One of the best characterised roles of Syp is its interaction with VAMP2 [45], a SNARE protein [67]. The complex between VAMP2 and Syp is formed at the trans-Golgi network (TGN), and the formation of this complex is necessary and sufficient to recruit VAMP2 to synaptic contacts. Disruption of this interaction results in diffuse localisation of VAMP2 along the axonal membrane [44]. Here we demonstrate that Aβ42 disrupts the interaction between VAMP2 and Syp both *in vitro* and in dissociated neurons (fig. 4). Two possible mechanisms could account for this disruption: either Aβ42 disrupts a signalling pathway that regulates the dynamics of this interaction at the TGN, or it competes with VAMP2 for binding to Syp. Our immunoprecipitation data demonstrate that Aβ42 directly competes with VAMP2 for binding to Syp supporting the latter hypothesis. We also observed that this disruption was more pronounced in less mature neurons (14 DIV) when synapses are not yet fully mature and there is increased traffic of structural components towards the developing synapses. In addition to correctly sorting VAMP2 at the synapse, Syp also regulates the retrieval kinetics of VAMP2 during endocytosis [63]. Thus, if Aβ42 competes with VAMP2 for binding to Syp at the synapse, it is likely that it will also affect SV recycling. Aβ42 induced changes to SV recycling has previously been reported. Kelly *et.al.* proposed that Aβ disrupts SV endocytosis, a phenotype consistent with reduced binding of Syp to VAMP2 in Aβ treated neurons [68]. Finally, it has also been reported that VAMP2 dissociates from Syp prior to exocytosis suggesting that Syp prevents VAMP2 from participating in the formation of the FPC [46], [69]. Furthermore, it has also been shown that Aβ induces an extensive depletion of the SV pool, indicative of an increased participation of SVs in neurotransmitter release [68]. Thus, we hypothesised that disruption of the interaction between Syp and VAMP2 would increase the availability of VAMP2, contributing towards an increase in the number of primed vesicles at the presynaptic membrane. We verified this by showing that fusion of primed SV in the presence of FM1-43FX results in an increase of endocytosed dye at the synapses of neurons incubated with Aβ42. In addition, the enhancement of single-shock fEPSPs by Aβ42 in synapses within hippocampal slices further suggested an increased availability of releasable SVs. What is more, the same effect was reproduced by naturally produced amyloid peptides demonstrating that this effect is independent of the peptide source. It is important to note that we were also able to reproduce the well-established disruption of LTP by Aβ42 under the same experimental conditions. In support of our results, several reports have shown an Aβ-dependent increase in the number of vesicles available at the presynaptic active zone [19], [28], [70] or that glutamate release is enhanced by Aβ peptides [71]. These results suggest that regulation of NT release at the pre-synaptic level could be an important aspect of the physiological role of Aβ. Furthermore, increasing evidence suggests that Aβ could exert its effects in synaptic plasticity by excessive activation of NMDARs. For instance, it has been shown that inhibition of LTP by Aβ can be via a mechanism involving excessive activation of extrasynaptic NMDARs as well as due to defects in glutamate uptake [29], [32], [33]. A key question that remains to be answered though is to what extent the interaction between Aβ and Syp that we report here, is necessary and sufficient to induce the observed increase in synaptic transmission, or to what extent this interaction is a contributing factor affecting the homeostasis of the presynaptic terminal. Although our data clearly demonstrate that Aβ is detected at the presynapse where it can interact with Syp affecting neurotransmitter release, its presynaptic effects are likely to be more complex. For instance, it has been shown that Aβ inhibits presynaptic P/Q Ca channels suppressing spontaneous synaptic activity [57], [72]. Interestingly, a series of studies demonstrated that Aβ can also have a positive effect on synaptic transmission in a concentration dependent manner. It has been shown that picomolar concentrations of Aβ42 enhance neurotransmitter release during HFS [19] and can also activate presynaptic α7-nAChRs [73]. Finally, Puzzo et.al showed that newly formed endogenous Aβ is essential for NT release from the presynaptic terminal [74], demonstrating further that at least in part, Aβ exerts its function at the pre-synapseThe, data presented here could contribute towards our understanding of the mechanism behind these observations. It is possible that the disruption of the Syp/VAMP2 complex contributes towards an increase in NT release, allowing VAMP2 to participate more readily in the formation of SNARE complexes deregulating NT release by increasing the amount of primed SVs. Moreover, following exocytosis, the ability of Syp to initiate SV endocytosis via its interaction with VAMP2 [62], [63] would be reduced. Such a mechanism can explain both the positive contribution of Aβ to NT release as well as the reported disruption of endocytosis and the depletion of SVs induced by Aβ [19], [68], [74]. Furthermore our data are also supported by findings that show a “bell shaped" relationship between Aβ and synaptic plasticity [28]. Consequently, long term exposure and/or high levels of Aβ42 would consistently deregulate glutamate release, disrupting LTP [33], [68], [70] and inducing excitotoxicity [75]. Finally, at later stages, increased release of Aβ42 from presynaptic sites [43] would induce additional effects at the postsynaptic membrane.

An increasing number of reports in the past years demonstrate that glutamatergic synaptic transmission via AMPA, NMDA and metabotropic glutamate receptors is crucial in the pathogenesis of AD. Targeting these receptors for AD therapy although beneficial comes with side effects. Thus, understanding the cellular mechanism behind the deregulation of glutamatergic neurotransmission might provide alternative therapeutic targets. Furthermore, in a recent publication, Puzzo et.al. pointed out that impairment of LTP at early stages of the disease is not necessarily linked to interference with the function of AMPAR or NMDAR and only increased concentrations of Aβ or prolonged exposure to the peptide showed a direct impairment of the receptors [25]. Our data further support this observation since we showed that brief exposure to Aβ42 at concentrations that are not known to directly interfere with the receptors, results in LTP impairment that is likely induced by pre-synaptic defects. Taken together, these data suggest that Aβ42 is involved in modulation of synaptic activity by interfering with multiple pre- and postsynaptic mechanisms in a concentration and time dependent manner.

Although our data showed the effect of Aβ42 on a cellular mechanism that can contribute to the deregulation of glutamatergic synaptic transmission, it is quite possible that we have demonstrated only one of several cellular events disrupted by Aβ. Nevertheless, our data make an important contribution in elucidating the pleiotropic function of Aβ. Understanding the causative events behind its toxicity is essential in order to design appropriate therapeutic strategies to target the symptoms of AD more efficiently.
